# P21-Activated Kinase 7 Mediates Cisplatin-Resistance of Esophageal Squamous Carcinoma Cells with Aurora-A Overexpression

**DOI:** 10.1371/journal.pone.0113989

**Published:** 2014-12-01

**Authors:** Shun He, Min Feng, Mei Liu, Shangbin Yang, Shuang Yan, Wei Zhang, Zaozao Wang, Chenfei Hu, Qing Xu, Lechuang Chen, Hongxia Zhu, Ningzhi Xu

**Affiliations:** 1 Laboratory of Cell and Molecular Biology and State Key Laboratory of Molecular Oncology, Cancer Hospital, Chinese Academy of Medical Sciences and Peking Union Medical College, Beijing, People's Republic of China; 2 Department of Pathology, West China Second University Hospital/West China Women's and Children's Hospital, Sichuan University, Chengdu, Sichuan, People's Republic of China; Institut de Génétique et Développement de Rennes, France

## Abstract

Aurora-A overexpression is common in various types of cancers and has been shown to be involved in tumorigenesis through different signaling pathways, yet how the deregulation affects cancer therapeutics remains elusive. Here we showed that overexpression of Aurora-A rendered esophageal cancer cells resistance to cisplatin (CDDP) by inhibiting apoptosis. By using an apoptosis array, we identified a downstream gene, p21-activated kinase 7 (PAK7). PAK7 was upregulated by Aurora-A overexpression at both mRNA and protein levels. Importantly, the expression levels of Aurora-A and PAK7 were correlated in ESCC primary samples. Chromatin immunoprecipitation (ChIP) assay revealed that binding of E2F1 to the promoter of PAK7 was significantly enhanced upon Aurora-A activation, and knockdown of transcription factor E2F1 decreased PAK7 expression, suggesting that Aurora-A regulated PAK7 through E2F1. Furthermore, we demonstrated that PAK7 knockdown led to increased apoptosis, and Aurora-A-induced resistance to CDDP was reversed by downregulation of PAK7, suggesting PAK7 was a downstream player of Aurora-A that mediated chemoresistance of ESCC cells to CDDP. Our data suggest that PAK7 may serve as an attractive candidate for therapeutics in ESCC patients with Aurora-A abnormality.

## Introduction

Aurora-A, a member of the Aurora kinase family, is an evolutionally conserved serine/threonine kinase that plays a vital role in maintaining proper chromosome segregation and cytokinesis during mitosis [1]. There have been extensive studies revealing that Aurora-A is also an important oncogene with frequent amplification and/or overexpression in multiple cancers [2]. It is reported that Aurora-A overexpression positively correlates with cancer stages in hepatocellular carcinoma, ovarian cancer, bladder cancer and head and neck cancer [3]–[6]. In the latter two cancer types, the aberrant expression of Aurora-A was also often found to be associated with metastasis. Mechanistic studies unravel that as a kinase, AURKA functions upon its substrates to activate a number of oncogenic proteins such as c-Myc [7], [8], and miRNAs such as miRNA-17-92 [9], as well as to inactivate tumor suppressor proteins such as p53 [10] and GSK3b [11], thereby promoting the pathogenesis of cancer. Inhibitors of Aurora-A are developed as potential anticancer drugs and some are in phase I and II clinical trials [12]. In a recent study, Aurora-A abnormality was detected in a number of patients of esophageal cancer [13], the fourth largest cancer type causing mortalities in China. Interestingly, it's reported that the Aurora-A inhibitor could enhance cisplatin (CDDP) -induced cell death in esophageal adenocarcinoma cells [12], therefore it's of great importance to examine how the abnormal expression of Aurora-A affects the treatment of these cancer patients.

P21-activated kinase 7 (PAK7) was first cloned and characterized a decade ago as a brain-specific kinase, which plays a role in controlling outgrowth of neuron cells as well as synaptic vesicle trafficking [14], [15]. Targeted disruption of PAK7, in combination of PAK6, which comes from the same family, leads to learning and locomotion defects in the knockout mice [16]. It is demonstrated that PAK7 is rarely expressed in the normal tissues except for brain; however, several recent studies reveal that PAK7 is aberrantly expressed in gastroenterological cancers, including colorectal and gastric cancers, making it a potential novel oncogene [17]–[20]. It's therefore of interest whether PAK7 is also involved in esophageal cancer development.

In this study we demonstrated that Aurora-A overexpression dramatically decreased the sensitivity of the esophageal cancer cells to the chemotherapy drug CDDP by inhibiting cell apoptosis. Further mechanistic study unraveled that Aurora-A induced expression of a downstream player PAK7, which was activated by transcription factor E2F1 through enhanced binding to the promoter. The elevation of PAK7 inhibited apoptosis of esophageal cancer cells, thereby contributing resistance to CDDP. Knockdown of PAK7 significantly increased sensitivity of tumor cells to CDDP treatment, unveiling a novel role of PAK7 in the treatment of esophageal cancers with Aurora-A overexpression.

## Materials and Methods

### Cell lines, Plasmids, siRNA, and transfection

Human ESCC cell line EC9706 was established [21] and provided by Prof. Mingrong Wang (Peking Medical College, Beijing, China) and was grown in DMEM (Invitrogen Life Technologies, Carlsbad, CA, USA). The Aurora-A expression plasmid pcDNA4-TO-Aurora-A was constructed as described previously [9]. AURKA kinase- dead (KD) mutant was created through K162M site-directed mutagenesis using QuikChange® Site-Directed Mutagenesis Kit (Stratagene, La Jolla, CA, USA). E2F1 siRNA sequence: sense: GCAUCCAGCUCAUUGCCAATT, antisense: UUGGCAAUGAGCUGGAUGCTT; PAK7 siRNA was provided as a TriFECTa Kit DsiRNA Duplex, which was customized by IDT(Coralville, IA, USA). Cells were transfected with Lipofectamine2000 (Invitrogen) according to the manufacturer's instruction. EC9706-Aur, EC9706-Aur-KD, and EC9706-P4 were selected with Zeocin (100 µg/ml) for 2 weeks. The small molecule inhibitor MLN8237 was purchased from Selleck Chemicals (Houston, TX, USA).

### RNA extraction, real-time PCR and apoptosis array

Total RNA was extracted with TRIZOL (Invitrogen) and reverse-transcribed to cDNA using MMLV Reverse Transcriptase (Promega). The quantitative real-time PCR of each sample was performed in triplicate on an ABI StepOne Plus (Applied Biosystems, CA) with SYBR Green PCR core reagents (Applied Biosystems, CA) according to the manufacturer's protocol. β-actin was used as a reference gene. The relative transcript levels in the treated sample compared to the control sample were determined as fold changes. PAK7 primers: sense: Atcaccttctcccagtattcca, antisense: ccgtgcttcattttcattacg. β-actin primers: sense: GGCGGCACCACCATGTACCCT, antisense: AGGGGCCGGACTCGTCATACT. For apoptosis array, appropriate amounts of cDNA were mixed with Realtime PCR master mix and loaded onto an apoptosis array (a 96-well plate containing a pre-designed primer pair specific for an apoptosis pathway gene or house-keeping (HK) gene in each well(SABiosciences, CA, USA). Data were analyzed as bellows: ΔCt was first calculated for each pathway-focused gene in each treatment group (ΔCt =  average Ct – average of HK genes' Ct), and then the ΔΔCt for each gene across two groups were calculated [ΔΔCt = ΔCt (group 2) - ΔCt (group 1)], where group 1 is the control and group 2 is the experimental. The fold-change for each gene from group 1 to group 2 was calculated as 2^−ΔΔCt^.

### Chromatin immunoprecipitation (ChIP)

ChIP assay was conducted with ChIP-ITTM Chromatin Immunoprecipitation Kits & Shearing Kits (Active Motif) according to the manufacturer's protocol. The antibodies for immunoprecipitation include an anti-E2F1 (sc-193x, Santa Cruz) antibody or mouse IgG (Active Motif). Primers specific for putative E2F-binding sites upstream of the PAK7 were: sense: CGCTCATGGACAGTCCTCCGA, antisense: GGTGAGAGGCAGAGCCACAGCTT.

### Western blotting

Cells were harvested and lysed in RIPA buffer. Protein concentrations were measured by BCA protein detection kit (Pierce, Rockford, IL, USA). Proteins were separated by SDS-PAGE and Transferred to EC membrane. Membranes were incubated with primary antibody at 4°C overnight, washed with TTBS (0.05% Tween 20 in Tris-buffered saline, TBS) and incubated with horseradish peroxidase conjugated secondary antibody (Zhongshan) for 1 h at RT and developed with Luminal Detection System (Santa Cruz Biotechnology Inc., Santa Cruz, CA, USA).

### FACS analysis

Cells were harvested and washed in cold sterile phosphate buffered saline (PBS). Annexin V and propidium iodide staining were carried out using the Annexin V-FITC Apoptosis Detection Kit (BD Biosciences) according to manufacturer's instructions. Analyses of apoptosis profiles were performed with Coulter Elite 4.5 Multicycle software.

### Immunohistochemistry

Esophageal cancer tissue array(cat#: ES481, ES482, ES807)were purcased from Yingchao Biotech, Xi'an, China. Specimens were incubated with anti-Aurora-A (Abcam; 1∶700) and anti-PAK7 (MBL; 1∶50) at 4°C overnight following the antigen retrieval in pH9.0 EDTA buffer in an autoclave for 2 min. All immunostaining experiments were assessed by an experienced pathologist. The protein expression within the cancer tissue was evaluated and categorized according to the percentagee and intensity of positive cells (tissues with no or weak staining in ≤5% of cells as “−/+”; with moderate staining in 6% to 25% of cells as “+”; with strong staining in 26% to 50% of cells as “++”; and with strong staining in ≥50% of cells as “+++”). Tumors were then further grouped into weak (−/+ and +) and strong (++ and +++) expression of each protein.

### MTT assay

EC9706-P4 and EC9706-Aur cells were plated at a density of 5000 cells/well into 96-well plates and then subjected to CDDP treatment at 10 µM for 55 hours. Media was aspirated, and 20 µL of MTT (5 mg/mL) was added to the cells. After 4 h of incubation (37°C, 5% CO_2_), the media were aspirated and 150 µL DMSO was added. The plates were placed on a shaking table at room temperature for 10 min. The cells were then measured by a Microplate Reader (Bio-Rad, Hercules, CA, USA) at a 570- nm wavelength.

### TUNEL assay

Cell apoptosis assay was performed as we described previously using the In Situ Cell Death kit (Roche Diagnosis) [22]. The treated cells were fixed for 1 hour with 4% paraformaldehyde in PBS (pH 7.4) and subsequently permeabilized for 2 min on ice with 0.1% sodium citrate solution containing 0.1% Triton X-100. After washing with PBS, cells were incubated with the TUNEL reaction mixture for 60 min at 37°C and subsequently, the NBT/BCIP solution. Analysis was performed under a light microscope.

### Statistical analysis

SPSS 15.0 for Windows (SPSS Inc.) was used for statistical analysis. Correlation between the Aurora-A expression levels and PAK7 expression profiles on a per-case basis was analyzed by Spearman's rank test. Values of p<0.01 were considered statistically significant.

## Results

### Aurora-A decreased esophageal cancer cells sensitivity to CDDP by inhibition of apoptosis

**Figure 1:**
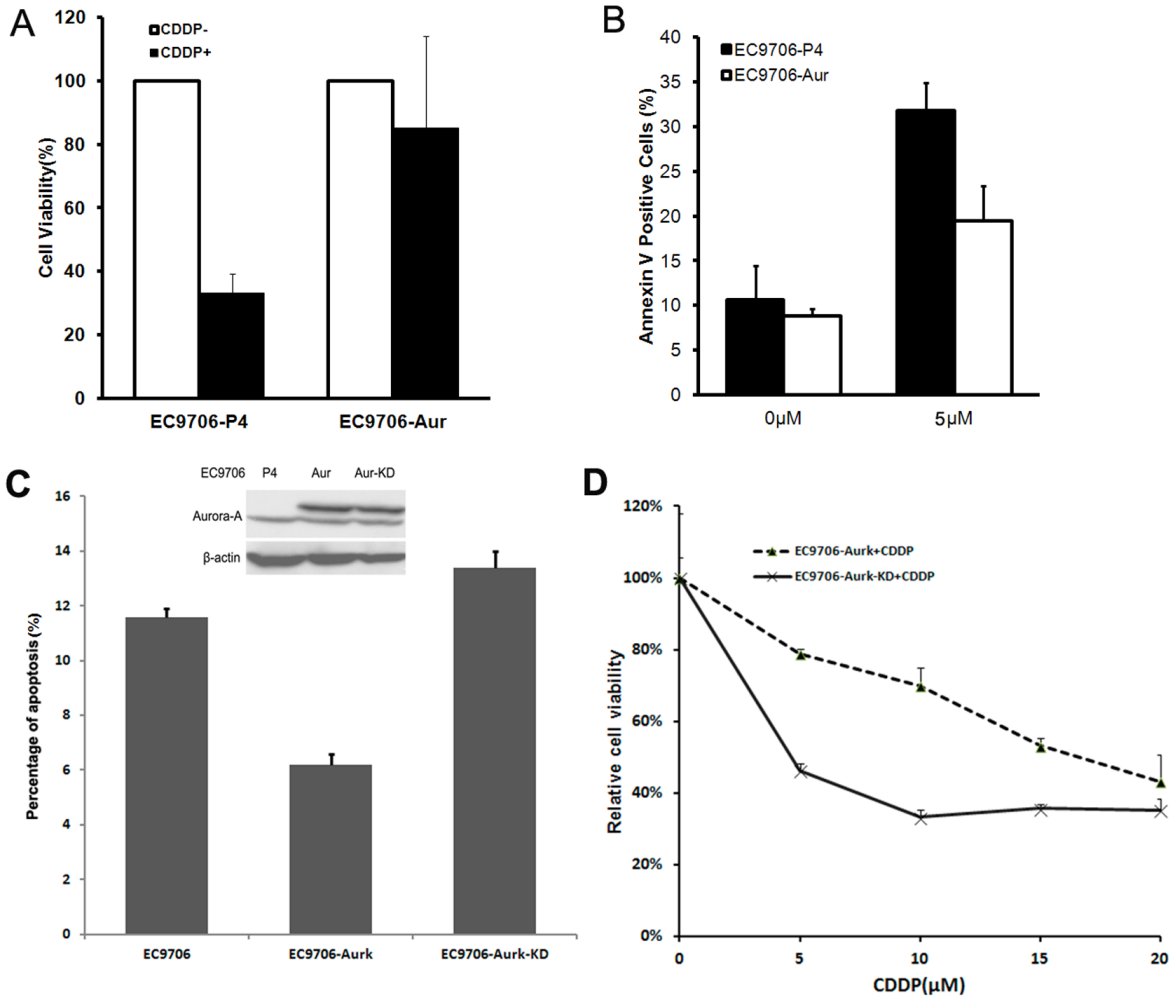
Aurora-A overexpression enhanced anti-apoptotic effects of CDDP in EC9706 cells. A: EC9706-P4 and EC9706-Aur cells were plated at a density of 5000 cells/well into 96-well plates and then subjected to CDDP treatment at 10 µM for 55 hours. The cell viablity was analyzed by MTT assay. B: EC9706-P4 and EC9706-Aur cells were treated with CDDP at 5 µM for 36 hours. Cells were harvested and stained for Annexin V/PI and subjected to flow cytometer for determination of apoptosis rates. C. EC9706, EC9706-Aurk and EC9706-Aurk-KD were treated with 10 µM CDDP for 48 hrs. Cells were harvested and fixed with 70% ethol for 1 hr, then cells were stained with PI and analyzed with Flow cell cytometer. D. EC9706-Aurk and EC9706-Aurk-KD cells were treated with different doses of CDDP and MTT assay was performed to determine the viability of the cells.

The abnormal expression of Aurora-A has been reported in esophageal cancers. To better understand the role of Aurora-A in esophageal cancers, we established Aurora-A overexpression cell line in EC9706 and explored how the high levels of Aurora-A would affect the response of the cancer cells to chemotherapeutic drugs. The cells stably expressing Aurora-A were treated with cisplatin, a drug commonly used in clinical treatment for esophageal cancers, for 24 hours and then cell viability was determined. As shown in Fig.1A, the viability of vector-transfected cells decreased dramatically upon exposure to CDDP, showing strong response to CDDP treatment; in the contrast, the cells with Aurora-A overexpression maintained viable at a significantly higher level after drug treatment, suggesting that the sensitivity of esophageal cancer cells to chemo drugs was attenuated by Aurora-A. Using Annexin V staining, we further detected the cell apoptosis induced by CDDP treatment. As shown in Fig.1B, compared with untreated cells, there was 3-fold increase for the vector-transfected ESCC cells which underwent apoptosis; however, there was only a modest increase of the apoptotic cells in cancer cells with Aurora-A overexpression, indicating that Aurora rendered resistance of esophageal cancer cells to chemo drugs by inhibition of cell apoptosis. To determine whether the kinase activity of Aurora-A is required in this process, we constructed a Kinase Dead (KD) Aurora-A and established a stable cell line EC9706-Aur-KD. We then treated both WT and KD cells with 10 µM CDDP. PI staining showed that EC9706-Aur had less percentage of apoptotic cells, while the cancer cells expressing kinase-dead Aurora-A showed comparable apoptosis rate to the parental cells (Fig 1C), indicating that kinase activity is important for chemoresistance induced by Aurora-A. We also compared the cell viability by MTT assay between EC9706-Aur and EC9706-Aur-KD cells upon different doses of CDDP treatment, and discovered that the kinase-dead cells (EC9706-Aur-KD) were more sensitive to CDDP treatment than their WT counterparts EC9706-Aur cells (Fig 1D), further implicating the involvement of kinase activity.

### Aurora-A induced PAK7 expression in esophageal cancer cells

**Figure 2:**
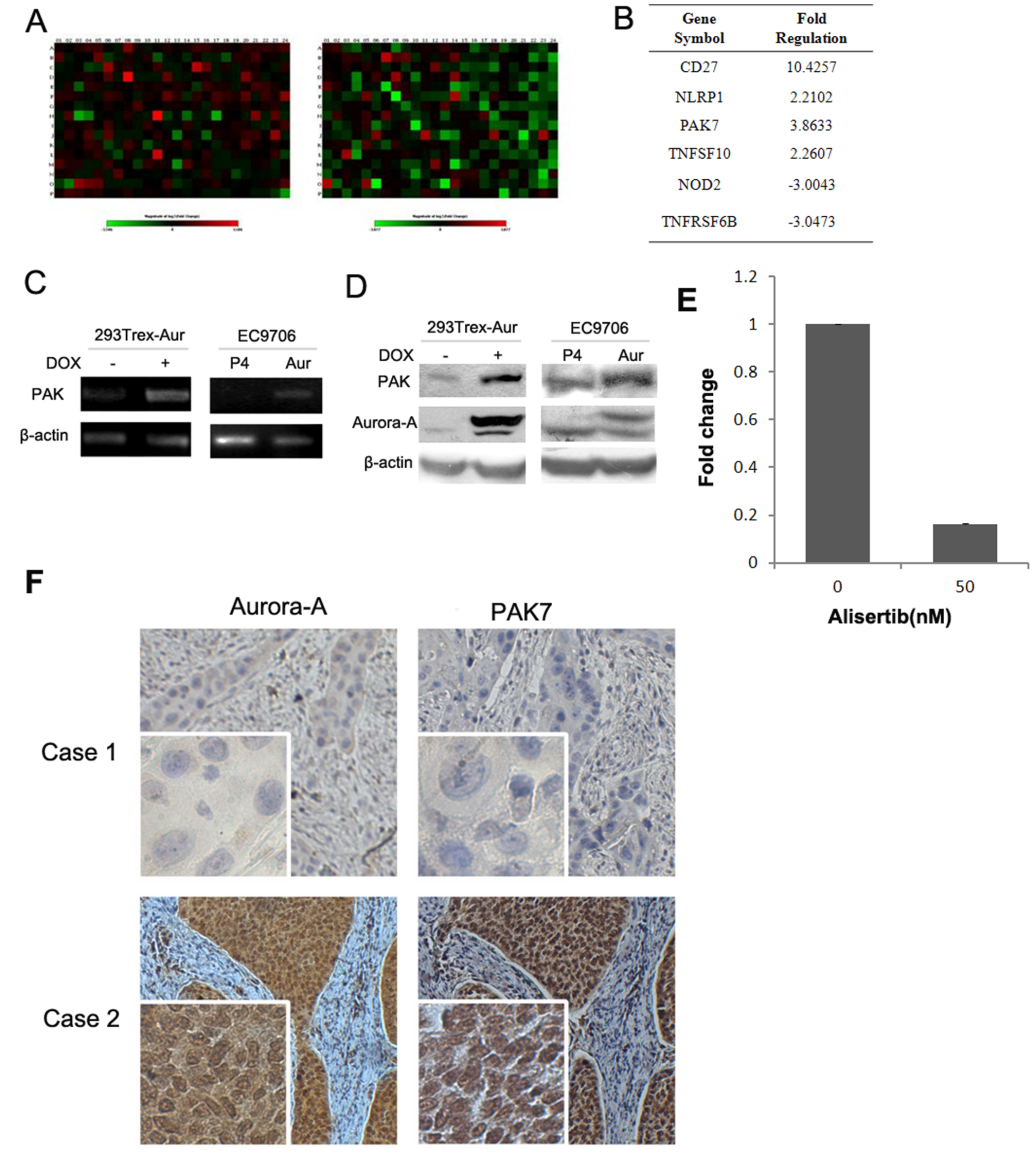
Aurora-A induced PAK7 expression in esophageal cancer cells. A: The heatmap of the array results. Left: 293TREX-Aur(DOX+/−); Right: EC9706-Aur/P4. B: Genes with upregulation or downregulation of more than 2-fold in both 293TREX-Aur and EC9706-Aur cells compared with control cells. The negative inverse results are reported as fold-downregulation. C. Aurora-A induced PAK7 mRNA expression. D. Aurora-A elevated PAK7 protein level. E. EC9706 cells were treated with 50 nM MLN8237 for 24 hrs and harvested for detection of PAK7 expression by Real-time PCR. F. Immunohistochemical analysis of Aurora-A and PAK7 expression in esophageal cancer samples. Representative cases of weak and strong staining for Aurora-A and PAK7 are shown. The original magnification: ×100 (pictures), and ×400 (insets).

To elucidate the molecular mechanism underlying apoptosis inhibition by Aurora-A, we took advantage of an apoptosis array, which detects the expression levels of a panel of apoptosis-related genes (including both pro- and anti- apoptosis genes) simultaneously. In addition to the esophageal cancer cell line, a previously reported 293TREX cell line capable of conditionally inducing Aurora-A was also employed to detect early changes induced by Aurora-A overexpression (Fig.2A). A total of 25 and 18 genes were found to be significantly upregulated in 293TREX cells and EC9706 cells after Aurora-A overexpression, respectively. Only 6 genes had the same alteration in both systems (Fig. 2B). Among them, PAK7 was shown to be increased 4-fold in both cell lines. Subsequent PCR and Western blot assays confirmed that PAK7 expression was induced by Aurora-A at both mRNA and protein level, validating the apoptosis array results (Fig.2C and 2D). Interestingly, expression level of PAK7 was decreased when the cells were treated with MLN8237, a small molecule inhibitor of Aurora-A, indicating that the regulation of PAK7 by Aurora-A requires the kinase activity (Fig 2E). To further verify the relationship between Aurora-A and PAK7, we also detected the expression levels of both proteins in 121 surgical specimens of primary esophageal cancers by immunohistochemistry (IHC). As shown in Table 1 and Fig.2F, in consistent with abnormal expression of Aurora-A, PAK7 overexpression was also detected in esophageal cancers, and more importantly, PAK7 expression was significantly positively correlated with Aurora-A level, underscoring the physiological relevance of the relationship between Aurora-A and PAK7 in ESCC.

**Table 1:** Summary of Aurora-A and PAK7 expression in human esophageal cancers.

Aurora-A Staining	PAK7 Staining		Total
	Weak	Strong	
Weak	23 (19%)	5(4%)	28(23%)
Strong	35(29%)	58(48%)	93(77%)
Total	58(48%)	63(52%)	121(100%)

The levels of Aurora-A and PAK7 expression were determined in 121 surgical specimens of primary esophageal cancers. The correlation was analyzed using a Spearman's rank correlation test, p<0.001.

### Aurora-A induced PAK7 expression through E2F1 regulation

**Figure 3:**
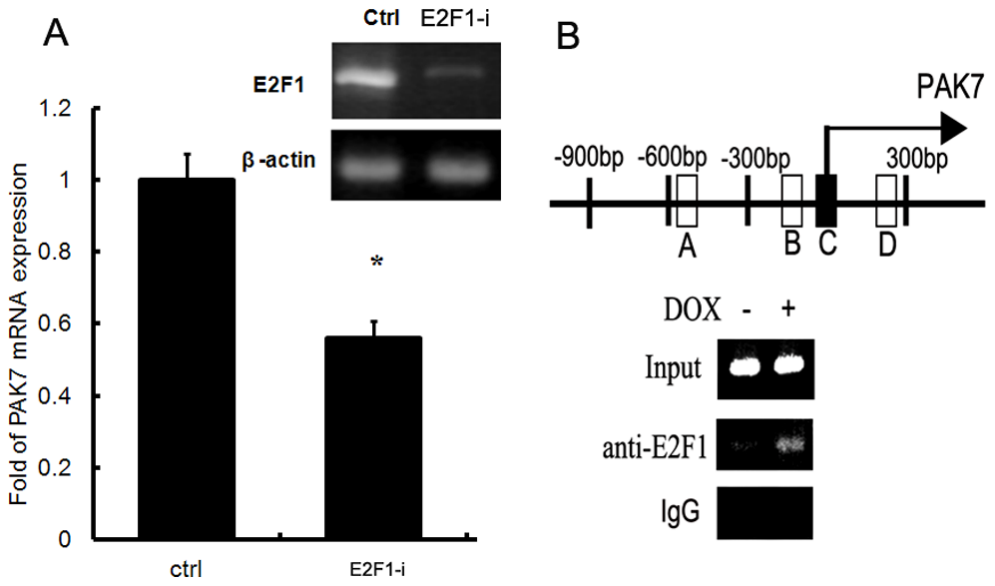
Aurora-A induced PAK7 expression through E2F1 regulation. A. Real-time PCR analysis of PAK7 mRNA expression in EC9706 cells transfected with E2F1 siRNA. B. Chromatins prepared from 293TREX-Aur cells treated with or without DOX were subjected to immunoprecipitation with either an anti-E2F1 antibody or mouse IgG. The precipitated DNA was detected by PCR assay. Input DNA of the two samples were subjected to PCR to ensure that equal amounts of total DNA were used in the immunoprecipitation.

Although we have demonstrated Aurora-A upregulated PAK7, yet the underlying mechanism remains elusive. We previously reported that Aurora-A was able to inhibit the degradation of E2F1, leading to the enhanced transactivity of this transcription factor and the upregulation of miR-17-92 expression [9]. We then explored whether PAK7 was one of E2F1 downstream targets that were upregulated due to the enhanced E2F1 transcriptional activity. We decreased E2F1 expression by siRNA knockdown and detected PAK7 expression level. As shown in Fig 3A, PAK7 level showed substantial decrease upon E2F1 downregulation, suggesting PAK7 was indeed regulated by E2F1. The promoter region of PAK7 was searched for E2F1 binding sequences, and four consensus sites were found. By using chromatin immunoprecipitation (ChIP), we demonstrated that Aurora-A overexpression significantly enhance E2F1 occupation on one E2F1 consensus site flanking PAK7 transcription starting site, while having no effects on the other three sites (Fig.3B). These data combined reveal that Aurora-A induced PAK7 via regulation of E2F1.

### Knockdown of PAK7 increased cell apoptosis in Aurora-A-overexpressing cancer cells

**Figure 4:**
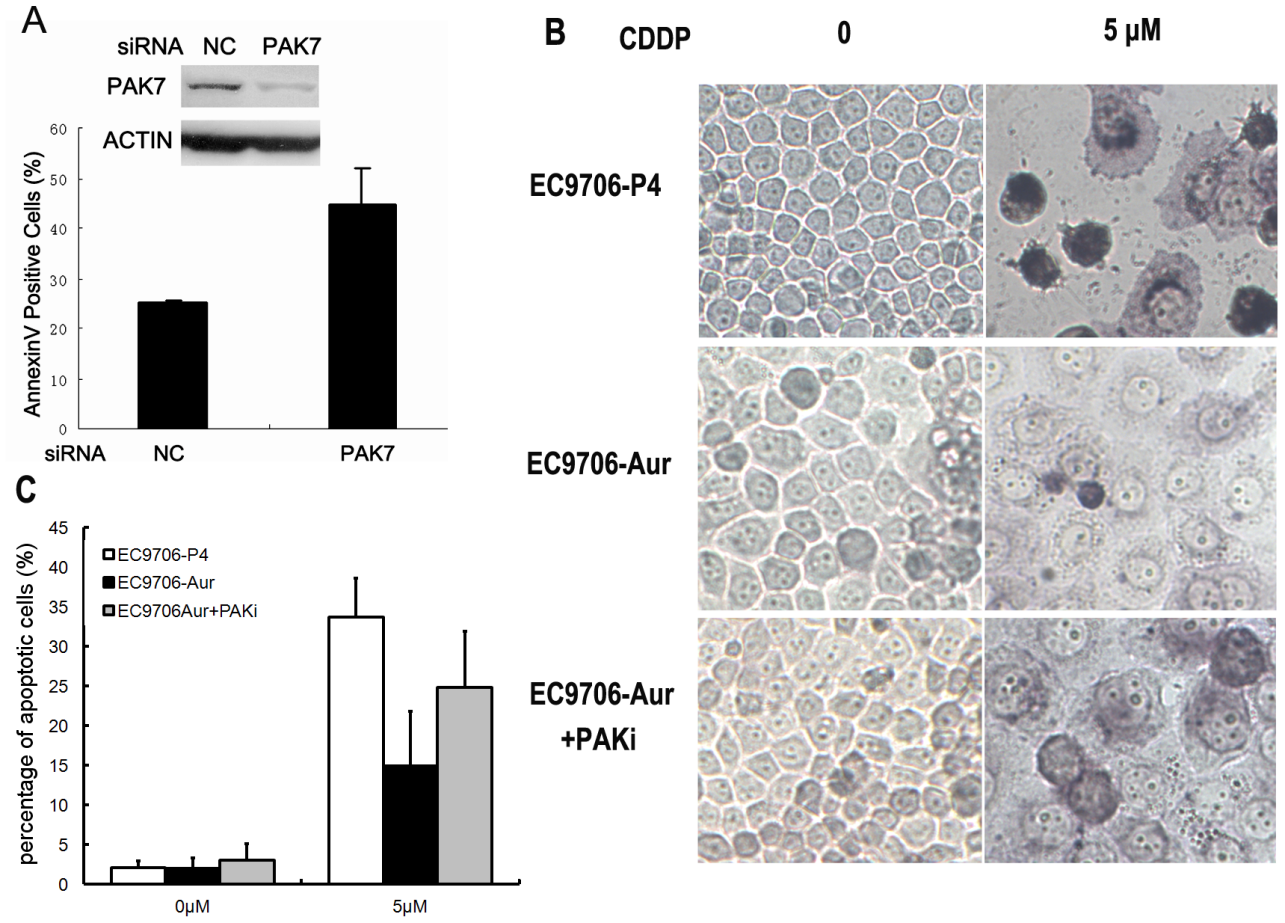
Knockdown of PAK7 increased cell apoptosis in Aurora-A-overexpressing cancer cells. A. EC9706-AURKA cells were treated with 80 nM PAK7 or Negative Control (NC) siRNA for 48 h and then exposed to 5 µM CDDP for 36 h. The cells were harvested and stained with AnnexinV/PI to determine apoptosis rates using flow cytometer. The efficiency of PAK7 knockdown was determined by Western blotting. B. TUNEL assay of EC9706 cells with different treatment. C. Percentage of TUNEL positive cells. Data represented the mean percentage from three independent experiments.

To further verify if Aurora-A-induced PAK7 in esophageal cancer cells mediates the apoptosis suppression by Aurora-A, esophageal cancer cells overexpressing Aurora-A were exposed to CDDP treatment after PAK7 expression was efficiently decreased by siRNA knockdown. The frequency of Annexin V positive cells (undergoing apoptosis) was about two-fold as high as that in control cells without PAK7 knockdown (Fig.4A). In consistent with this, another apoptosis detection experiment, TUNEL, also showed that PAK7 knockdown was able to significantly reverse the apoptosis resistance rendered by Aurora-A, further demonstrating that Aurora-A induced drug resistance is at least partly mediated by PAK7 (Fig. 4B and 4C).

## Discussion

Esophageal cancer is one of the most aggressive cancers and ranks sixth among causes of cancer death worldwide. A majority of esophageal cancer cases are squamous cell carcinoma (ESCC) [23], [24]. Chemotherapy regimens are recommended for ESCC patient's treatment by NCCN Guidelines, and CDDP is one of the cytotoxicity drugs that are most often used in the treatment of ESCC. However, due to the complexity of the biology of tumorigenesis, all ESCC patients are not having good response to this regimen, urging an exploration of the screening of the chemosensitivity of patients before treatment. Here we demonstrated that Aurora-A overexpression in ESCC renders cancer cells resistance to CDDP by antagonizing apoptosis. This effect was mediated through PAK7, which is upregulated transcriptionally by E2F1. Knockdown of PAK7 led to increase of sensitivity of Aurora-A overexpressing ESCC cells to CDDP, implicating that additional strategies, i.e., PAK7 targeting, are required along with CDDP-based chemotherapy to achieve desirable therapeutic effects in the subsets of ESCC patients with Aurora-A overexpression.

PAK7 is seldom expressed in the normal tissues other than brain. Here we report that PAK7 expression is elevated in esophageal cancer, confirming the involvement of this kinase in the tumor development. It is consistent with several recent studies revealing that PAK7 is also aberrantly expressed in a various types of cancers, including colorectal, gastric, epithelial ovarian and breast cancers [17]–[20]. However how PAK7 is regulated in tumor cells remain largely unknown. Here, for the first time to our best knowledge, we elucidate that PAK7 is a direct target of the transcription factor E2F1, which upon activation of Aurora-A, binds to promoter of PAK7 and transactivates PAK7 expression. Such regulation between Aurora-A and PAK7 is further supported by the correlation of the expression levels of both proteins in primary esophageal cancer samples.

Although the exact role of PAK7 in tumorigenesis is still not very clear, a lot of evidence suggests that its oncogenic activity is attributable to the ability of apoptosis inhibition. Cotteret et al reported PAK7 localizes to mitochondria and inhibits apoptosis through BAD phosphorylation, and nucleocytoplasmic shuttling regulates this anti-apoptosis function [25], [26]. The unique property implicates that this gene could play an important role in cancer therapeutics in which the cancer drugs eradicate tumor cells by inducing apoptosis. Indeed, Wang et al reported that overexpression of PAK7 inhibited camptothecin-induced apoptosis by inhibiting the activity of caspase-8 in colorectal carcinoma cells [27]. In this study we discovered that in the context of esophageal cancers where abnormal expression of Aurora-A induced cell resistance to CDDP treatment, PAK7 upregulation and thereby inhibition of apoptosis, is of vital importance, suggesting that PAK7 may be a promising therapeutic target candidate in cancer treatment. During the preparation of our manuscript, another group also demonstrates that relatively higher PAK7 expression is observed in epithelial ovarian cancer patients who show chemoresistance to paclitaxel treatment, while down-regulation of PAK7 restores ovarian cancer cells chemosensitivity to paclitaxel [19], underscoring the pivotal role of PAK7 in cancer chemotherapy.

In summary, in this study we discovered a novel relationship between Aurora-A and PAK7, and unveiled a molecular mechanism underlying the CDDP resistance of esophageal cancer cells, providing a novel therapeutic strategy for esophageal cancer treatment.
